# Pentalogy of Cantrell: Prenatal Diagnosis, Delivery, and Immediate Postnatal Surgical Repair

**DOI:** 10.21699/jns.v6i2.503

**Published:** 2017-04-15

**Authors:** Edward Araujo Júnior, Milene Carvalho Carrilho, Bruno Rodrigues Toneto, José Cícero Stocco Guilhen

**Affiliations:** 1 Department of Obstetrics, Paulista School of Medicine - Federal University of São Paulo (EPM-UNIFESP), São Paulo-SP, Brazil; 2 Discipline of Cardiovascular Surgery, Department of Surgery, Paulista School of Medicine - Federal University of São Paulo (EPM-UNIFESP), São Paulo-SP, Brazil

**Keywords:** Pentalogy of Cantrell, Prenatal diagnosis, Delivery, Postnatal Surgery

## Abstract

Pentalogy of Cantrell (PC) is a congenital anomaly characterized by a defect in the lower sternum, anterior diaphragm, and anterior abdominal wall; ectopia cordis; and congenital heart disease. It is a very rare congenital anomaly and the prenatal diagnosis is possible in the beginning of second trimester of pregnancy using the conventional ultrasonography. The prognosis is poor with high rates of perinatal mortality. We present a case report of prenatal diagnosis of PC at 22 weeks and 3 days of gestation. We emphasize the prenatal care follow up in a tertiary reference center, the parental counseling, the planning of delivery, and the management of newborn by a multidisciplinary team, including the description of immediate postnatal surgical repair.

## Introduction

Pentalogy of Cantrell (PC) is a congenital anomaly characterized by a defect in the lower sternum, anterior diaphragm, and anterior abdominal wall; ectopia cordis; and congenital heart disease (CHD) that was first described in 1958 by Cantrell [1]. Prenatal diagnosis of PC can be made in the first trimester using transvaginal color Doppler sonography [2]. However, most cases are diagnosed early in the second trimester, when ectopia cordis associated with omphalocele is observed [3]. The prevalence of PC is 1:65,000 live births, with a 37% survival rate [4]. Surgical treatment should be performed at the earliest to prevent cardiac trauma; however, surgery depends on the size of the sternal defect, and the use of autologous tissues is preferable [5]. In this article, the authors present a case of PC diagnosed in the second trimester, focusing on the main ultrasound and fetal echocardiography findings as well as on delivery planning and surgical correction in the postnatal period. 

## Case report

A primigravida patient aged 42 years was referred to Department of Obstetrics at 22 weeks and 3 days of gestation with obstetric ultrasonography findings suggestive of ectopia cordis. The patient presented hypothyroidism and chronic hypertension, with no family history of birth defects or CHD. Fetal echocardiography confirmed the presence of ectopia cordis associated with a small ventricular septal defect (VSD) measuring 2.7 mm (Fig.1). In addition to ectopia cordis, a fetal morphology ultrasound demonstrated the presence of omphalocele with liver herniation, without other malformations. In light of the echocardiographic and ultrasound findings, the diagnosis of PC was suggested. The patient was followed up, with genetic counseling, psychological support, and serial obstetric ultrasonography for evaluation of fetal growth. At week 34, the patient presented premature rupture of membranes, with spontaneous onset of labor. Considering the presence of ectopia cordis and the breech position of the fetus, a cesarean delivery was selected. The delivery was uneventful, and a female child weighing 2,430 g, with Apgar scores of 8 and 9 at 1 and 5 min, respectively, was born (Fig. 2).

Within 11hr of life, the authors opted for surgical correction, which comprised opening of the remnant of the manubrium, total thymectomy, wide opening of both the pleura, resection of the remaining pericardium, bilateral deinsertion of the last three costal arches, and bilateral wide release of a skin flap from the midline to the anterior axillary line. Using these maneuvers, the heart could be accommodated into the chest cavity; the epigastric omphalocele was corrected with abdominal flap rotation and coverage of the diaphragm with a silicon mesh. Chest tubes were bilaterally placed; an orogastric and urinary catheter was placed, and phlebotomy was performed. Antibiotic therapy was initiated with gentamicin and ampicillin; adrenaline was administered for improving the cardiac output. On the 3^rd^ postoperative day, the patient developed anuria unresponsive to diuretics; peritoneal dialysis was initiated, and vasoactive drugs such as milrinone and noradrenaline were administered. On the 7^th^ postoperative day, the patient progressed with anasarca, and a total abdominal ultrasound showed signs of nephrocalcinosis and ascites; an ascitic fluid culture showed bacterial peritonitis. On the 8^th^ postoperative day, a left pneumothorax with hemodynamic repercussion was diagnosed and promptly drained; parenteral nutrition was then introduced. The patient developed metabolic acidosis and worsening of hemodynamic parameters and died on the 17^th^ postoperative day because of septic shock. 

## Discussion

There are no studies assessing the best mode of delivery in cases of PC because this is a rare congenital anomaly with poor prognosis for which pregnancy termination is indicated in many countries. In a series of 13 cases of PC, the mortality rate was 84.6%, with a mean age at death of 3.6 months and the main cause of death being heart failure (31%), hypoxia (23%), or perioperative death (23%) [6]. The authors opted for an early correction of ectopia cordis because of the presence of a minor CHD. In a series of 13 cases of PC, six patients received some type of surgical treatment and five underwent cardiac surgery [6]. Review of literature shows that autologous tissues and biocompatible materials have been used for chest wall closure [7,8]. The results are obviously better in patients with minor defects [7]. 

## Footnotes

**Source of Support:** None

**Conflict of Interest:** None

## Figures and Tables

**Figure 1:**
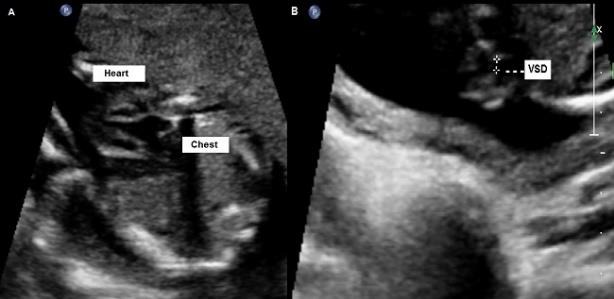
A. Axial plan of the fetal thorax showing ectopia cordis. B. Axial plan of the fetal thorax showing ventricular septal defect.

**Figure 1:**
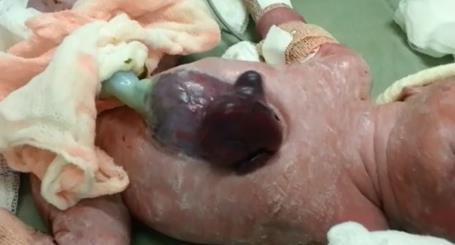
Photo of the newborn showing omphalocele and ectopia cordis.
